# Expression of the translocator protein (TSPO) from *Pseudomonas fluorescens* Pf0-1 requires the stress regulatory sigma factors AlgU and RpoH

**DOI:** 10.3389/fmicb.2015.01023

**Published:** 2015-09-24

**Authors:** Charlène Leneveu-Jenvrin, Emeline Bouffartigues, Olivier Maillot, Pierre Cornelis, Marc G. J. Feuilloley, Nathalie Connil, Sylvie Chevalier

**Affiliations:** Laboratory of Microbiology Signals and Microenvironment, University of RouenEvreux, France

**Keywords:** TSPO, AlgU, *rpoH*, osmolarity, temperature

## Abstract

The translocator protein (TSPO), previously designated as peripheral-type benzodiazepine receptor, is an evolutionary conserved protein that is found in many Eukarya, Archae, and Bacteria, in which it plays several important functions including for example membrane biogenesis, signaling, and stress response. A *tspo* homolog gene has been identified in several members of the *Pseudomonas* genus, among which the soil bacterium *P. fluorescens* Pf0-1. In this bacterium, the *tspo* gene is located in the vicinity of a putative hybrid histidine kinase-encoding gene. Since *tspo* has been involved in water stress related response in plants, we explored the effects of hyperosmolarity and temperature on *P. fluorescens* Pf0-1 *tspo* expression using a strategy based on *lux*-reporter fusions. We show that the two genes Pfl01_2810 and *tspo* are co-transcribed forming a transcription unit. The expression of this operon is growth phase-dependent and is increased in response to high concentrations of NaCl, sucrose and to a D-cycloserine treatment, which are conditions leading to activity of the major cell wall stress responsive extracytoplasmic sigma factor AlgU. Interestingly, the promoter region activity is strongly lowered in a *P. aeruginosa algU* mutant, suggesting that AlgU may be involved at least partly in the molecular mechanism leading to Pfl01_2810-*tspo* expression. *In silico* analysis of this promoter region failed to detect an AlgU consensus binding site; however, a putative binding site for the heat shock response RpoH sigma factor was detected. Accordingly, the promoter activity of the region containing this sequence is increased in response to high growth temperature and slightly lowered in a *P. aeruginosa rpoH* mutant strain. Taken together, our data suggest that *P. fluorescens tspo* gene may belong at least partly to the cell wall stress response.

## Introduction

Tryptophan-rich sensory protein/peripheral-type benzodiazepine receptor (TspO/MBR) domain-containing proteins also called translocator proteins (TSPOs) are membrane anchored proteins that have already been described in several bacteria, Archae, and Eukarya (Lacapere and Papadopoulos, 2003; Papadopoulos et al., 2006; Fan et al., 2012; Leneveu-Jenvrin et al., 2014). The best characterized member of this family is the mammalian 18-kDa TSPO protein, which is essentially located in the mitochondrial outer membrane associated with the voltage-dependent anion channel (VDAC). It has been involved in many fundamental physiological functions in mammals, including cell growth and proliferation, immunomodulation, apoptosis, and adaptation to oxidative stress (Papadopoulos et al., 2006; Guo et al., 2015), as well as in various activities related to mitochondrial physiology, including cholesterol import and steroid hormone biosynthesis (Papadopoulos et al., 1997; Miller and Bose, 2011; Issop et al., 2013). In mammals, TSPO is expressed in almost every tissue type, but is particularly enriched in steroidogenic and some cancer cells (Hardwick et al., 1999; Galiegue et al., 2003). TSPO expression dysregulation has been correlated to several diseases, including cancer (Batarseh and Papadopoulos, 2010), neuronal damage, neurodegeneration, and inflammation (Harberts et al., 2013; Dickens et al., 2014). In addition, a second mammalian TSPO isoform that is involved in erythropoiesis has been described (Fan et al., 2009). Overall, considering these important functions and perspectives in mammals, TSPO has been the focus of multiple studies during these last 30 years. However, the precise physiological function of TSPO in mammals remains a controversial, elusive, and currently intensively debated question (Batoko et al., 2015; Gut et al., 2015; Li et al., 2015; Milenkovic et al., 2015; Papadopoulos et al., 2015; Selvaraj and Stocco, 2015). For example, while TSPO was shown to bind many ligands including cholesterol (Li and Papadopoulos, 1998) and porphyrins (Verma et al., 1987), its function as cholesterol or porphyrin translocator is yet still unclear (Morohaku et al., 2013; Batoko et al., 2015; Gut et al., 2015; Li et al., 2015; Tu et al., 2015). Very recently, it has been proposed that TSPOs may be involved at least partly in complex homeostasis signaling mechanisms, related in particular to oxidative stress (Batoko et al., 2015).

TSPO-related proteins were also identified in plants (Corsi et al., 2004; Lindemann et al., 2004; Frank et al., 2007). In the moss *Physcomitrella patens*, three genes encode *tspo* homologs. One of these, PpTSPO1, is up-regulated by abiotic stress at the transcriptional level, and a knockout mutant of this gene led to increased sensitivity to salt stress (Frank et al., 2007). In angiosperm species such as *Arabidopsis thaliana*, a single gene encodes a TSPO-related protein (AtTSPO). AtTSPO is transiently expressed in dry seeds and can be induced in vegetative tissues by osmotic and salt stresses or by abscisic acid (ABA) treatments, suggesting that AtTSPO is specifically induced by water-related stress (Guillaumot et al., 2009a,b; Balsemão-Pires et al., 2011). Constitutive expression of this protein has been found to be detrimental to plant cells and seems to increase their sensitivity to ABA, suggesting that AtTSPO may be required transiently in an ABA-dependent stress response (Guillaumot et al., 2009a,b).

Bacterial TSPO appears to be widely distributed in eubacteria since a *tspo* homolog gene has been predicted in 97 different eubacterial species or strains encompassing most of the different taxonomic groups (Chapalain et al., 2009). The best characterized member of the *tspo* family is found in the a proteobacterium *Rhodobacter sphaeroides* (RsTspO). RsTSPO is localized in the bacterial outer membrane and its expression is induced by oxygen (Yeliseev and Kaplan, 1999). Under conditions of high oxygen, TSPO was shown to function as a negative transcriptional regulator of genes involved in photo-pigment biosynthesis, likely controlling the eﬄux of an intermediate in the heme biosynthesis pathway (Yeliseev and Kaplan, 2000; Zeng and Kaplan, 2001). A similar mechanism was proposed for the regulation of a nutrient deprivation-induced (*ndi*) locus in the endosymbiotic soil bacteria *Sinorhizobium meliloti* (Davey and de Bruijn, 2000).

Interestingly, a *tspo* homolog gene has been identified in several members of the *Pseudomonas* genus, among which *P. fluorescens* Pf0-1 (Chapalain et al., 2009; Leneveu-Jenvrin et al., 2014). *P. fluorescens* Pf0-1 is a common psychrotrophic Gram-negative inhabitant of soil and rhizosphere, and in its genome the *tspo* gene is located in the vicinity of a putative hybrid histidine kinase-encoding gene (Leneveu-Jenvrin et al., 2014). In this strain, both genes are predicted to form an operonic structure (Winsor et al., 2011). This study aims at giving new insights into *tspo* expression of *P. fluorescens* Pf0-1 using a strategy based on *lux*-reporter fusions. Since *tspo* has been involved in water stress related-response in plants, we explored the effects of such stresses on *P. fluorescens* Pf0-1 *tspo* expression.

## Materials and Methods

### Bacterial Strains and Culture Conditions

The strains used are listed in **Table 1**. *P. fluorescens* strains were grown at 17, 28, or 32°C on a rotary shaker (180 rpm) in Luria-Bertani (LB) broth supplemented or not with additional NaCl 171 (LB171N) or 342 (LB342N) mM, sucrose 342 (342S) or 684 (LB684S) mM, or D-cycloserin 50 μg ml^-1^. For each condition, a pre-culture was inoculated at an initial optical density at 600 nm (OD_600_) of 0.08 in LB medium. Stationary phase cells grown in LB medium were diluted at 1/100 (v/v) in LB or in modified LB, and their growth was followed in covered white 96-well OptiPlates with a flat transparent bottom (BD Falcon, San Jose, CA, USA), unless otherwise specified. Bacterial growth was monitored throughout time using a multimode plate reader (Flex-Xenius XM; SAFAS, Monaco). When required (for plasmid maintenance), *P. fluorescens* Pf0-1 or *P. aeruginosa* PA01 was grown at 28 or 37°C, respectively, in LB liquid cultures in the presence of Gm (50 μg ml^-1^), or on LB agar (1.5%) plates containing Gm (50 μg ml^-1^).

**Table 1:** Bacterial strains and plasmids used in this study.

Strain or plasmid	Characteristics	Reference
**Strains**		
*Pseudomonas fluorescens*		
Pf0-1	Wild type	Silby et al., 2009
*P. aeruginosa*		
PAO1	Wild type	Woodruff and Hancock, 1989
PAOU	PAO1Δ*algU*	Bazire et al., 2010
*rpoH*	Transposon mutant *rpoH*::IS;phoA/hah-Tc^r^	University of Washington mutant library (Jacobs et al., 2003)
**Plasmids**		
pAB133	Gm^r^; pBBR1MCS-5-based cloning vector containing the promoterless *luxCDABE* operon.	Bazire et al., 2005
pTSPO	Gm^r^; pAB133-based plasmid with the 253-bp *tspo* upstream fragment at the *Sac*I and *Spe*I sites.	This study
pHK-TSPO	Gm^r^; pAB133-based plasmid with the 227-bp Pfl01_2810-*tspo* upstream fragment at the *Sac*I and *Spe*I sites	This study
pHK-TSPO-88	Gm^r^; pAB133-based plasmid with the 89-bp Pfl01_2810-*tspo* upstream fragment at the *Sac*I and *Spe*I sites.	This study
pHK-TSPO-157	Gm^r^; pAB133-based plasmid with the 70-bp Pfl01_2810-*tspo* upstream fragment at the *Sac*I and *Spe*I sites	This study
pHK-TSPO-227	Gm^r^; pAB133-based plasmid with the 69-bp Pfl01_2810-*tspo* upstream fragment at the *Sac*I and *Spe*I sites	This study

### Construction of Transcriptional Fusions

**FIGURE 1:**
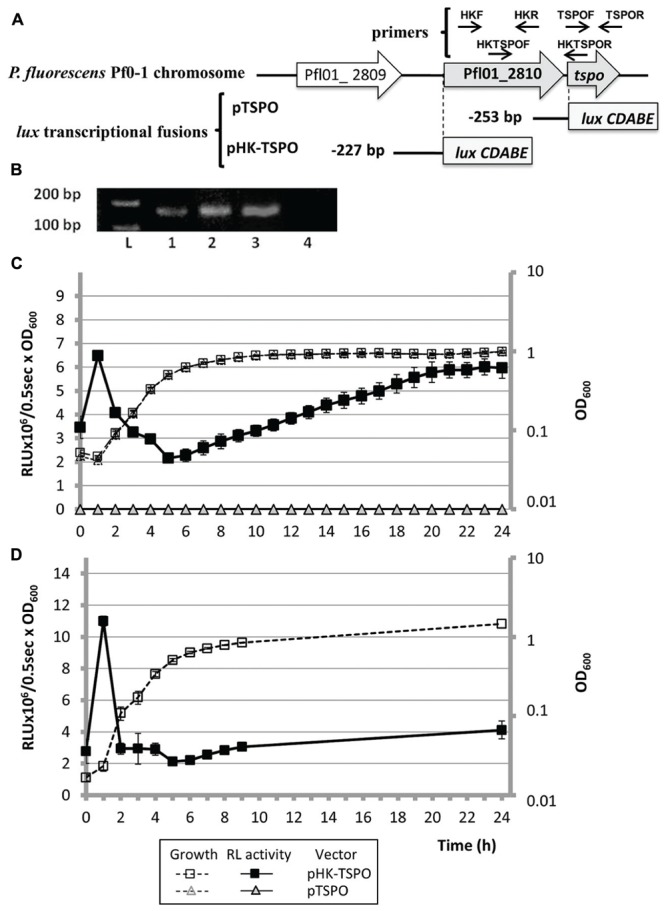
**The *tspo* gene (Pfl01_2811) of *Pseudomonas fluorescens* Pf0-1 forms an operonic structure with Pfl01_2810 that is expressed transiently in the bacterial growth course. (A)** Schematic representation of the genomic environment of *tspo* and of transcriptional fusions pTSPO and pHK-TSPO. Gray bars represent the beginning of the *luxCDABE* reporter cassette of the promoterless pAB133 vector. The position of the studied promoter region is indicated relatively to the translational initiation start of each ORF. **(B)** Co-transcription of Pfl01_2810 and *tspo* by reverse transcription-PCR (RT-PCR) assay. RT-PCR was assayed on *tspo* (1), Pfl01_2810 (2) and on the putative operonic structure Pfl01_2810-*tspo* (3), using primers located into *tspo* (1) or Pfl01_2810 (2) ORFs, or into both *tspo* and Pfl01_2810 ORFs (3). RT-PCR achieved on total RNA did not lead to a PCR fragment (line 4, negative control). L: Ladder 1kb+ (Biorad^®^), primers sequences are given in **Table 2**. **(C)** Growth curves in microtitre wells in Luria-Bertani (LB) medium at 28°C and relative luminescence (RL) activity of pTSPO and pHK-TSPO in *P. fluorescens* Pf0-1. **(D)** Growth curves in erlenmeyer in LB medium at 28°C and RL activity of pHK-TSPO in *P. fluorescens* Pf0-1.

To monitor *tspo* transcription, *tspo* and *Pfl01_2810-tspo* promoter regions were fused to the promoter-less *luxCDABE* cassette in the replicative low-copy-number pAB133 vector (Gm^r^) (Bazire et al., 2005). The promoter regions of interest are located in **Figures 1A** and **5A**, relatively to the *tspo* and *Pfl01_2810* genes. The primer sequences, which were used for PCR amplifications, are shown in **Table 2**. The *Sac*I*-Spe*I-digested PCR products were inserted into pAB133, yielding pTSPO, pHK-TSPO, pHK-TSPO-227, pHK-TSPO-157, and pHK-TSPO-88. The inserts were verified by DNA sequencing (Beckman Coulter Genomics).

**Table 2:** Primers used in this study.

Primer	Name	Sequence (5′-3′)^∗^
HKF		GCCCTGCTCAACCTGTGTAT
HKR		CCTAGCGGTTTGGTGGTAAA
TSPOF		AACAAGCCGAAATTCACACC
TSPOR		CACAGCAGGACGAGAATGAG
HKTSPOF		CGGGTCAGTGAATTGATGTG
HKTSPOR		GGTGTGAATTTCGGCTTGTT
F1	pTSPO	taataagagctcAGCGCATGAGCATGATGTA
R1	pTSPO	taataaactagtCCGACAGGTGCAGTCAAT
F2	pHK-TSPO	taataagagctcATGAACGGTCGACAACTGG
R2	pHK-TSPO	taataaactagtTTCTTACTCCTTGGCCGC
F3	pHK-TSPO-227	taataagagctcAGCGCATGAGCATGATGTAC
R3	pHK-TSPO-227	taataaactagtCGTTGCCAGAGTGCCTGT
F4	pHK-TSPO-157	taataagagctcCGGCCAGGCATTGTTGG
R4	pHK-TSPO-157	taataaactagtGTCGTATCGGGCTAGCACT
F5	pHK-TSPO-88	taataagagctcCTTGCTCCATCAGTGAAACTTAA
R5	pHK-TSPO-88	taataaactagtACAGGTGCAGTCAATATCGG
16SF		CTGGTAGTCCACGCCGTAAAC
16SR		CCAGGCGGTCAACTTAATGC
Pfl012810F		AGCTGATGCCGCTCTACGA
Pfl012810R		GTACGG-TCGCGGAGAATTTTC

*^∗^All primers used in this study were synthesized by Eurogentec and are based on *P. fluorescens* Pf0-1 genome sequence (http://www.pseudomonas.com). Nucleotides in lowercase are not complementary to the target sequence. Underlined nucleotides indicate restriction endonuclease sites inserted within primer sequences.*

### Bioluminescence Assays

*Pseudomonas fluorescens* or *P. aeruginosa* strains containing pAB133-derived plasmids were grown in covered, white 96-well OptiPlates with a flat transparent bottom (BD Falcon, San Jose, CA, USA). Bioluminescence and absorbance were simultaneously measured throughout bacterial growth using a multimode plate reader (Flex-Xenius XM; SAFAS, Monaco). The bioluminescence values (in relative light units [RLU] ⋅ 0.5 s^-1^) were divided by the absorbance values at 600 nm, yielding to relative bioluminescence values (in RLU ⋅ 0.5 s^-1^ ⋅ A_600_^-1^). The relative luminescence (RL) values of the negative-control strain *P. fluorescens or P. aeruginosa* harboring the empty vector pAB133 were subtracted from those of the studied *Pfl01_2810-tspo* promoter regions, as previously described (Bouffartigues et al., 2012). Each set of experiments was performed at least three times.

### Total RNAs Extraction

Total RNA extraction was achieved by the hot acid-phenol method. Briefly, cells were lysed and RNAs were extracted three times with an equal volume of acidic hot phenol and once with chloroform. After ethanol precipitation, RNAs were air dried and dissolved in water. Contaminating DNA was removed from total RNA by using 10 U of RNase-free DnaseI (New England BioLabs France) in a 50 μl mixture containing 6.25 mM MgCl_2_ and approximately 3 μg μl^-1^ of total RNA. The reaction mixture was incubated 30 min at 37°C, and the DNase I was then inactivated by adding 1 μl of 0.5 M EDTA to the mixture and incubating the mixture for 10 min at 65°C. The concentration was determined by measuring the absorbance at 260 nm. The quality of the RNA was then checked on a 2% agarose gel prior to use. Each RNA extraction was tested by PCR for the absence of contaminating DNA prior reverse transcription assays, and was performed in triplicate.

### RT-PCR/qRT-PCR Experiments

The primers used for reverse transcription-PCR (RT-PCR) or quantitative reverse transcription-PCR (qRT-PCR) are given in **Table 2**. Reverse transcription was used to assay co-transcription of genes belonging to the same operonic structure. Briefly, 10 ng of purified and controlled total RNAs were subjected to a one step RT-PCR assay according to the manufacturer instructions (Life Science, Roche, USA), before gel electrophoresis. qRT-PCR assays were achieved according to Gicquel et al. (2013), 16S rRNA was used as an endogenous control for qRT-PCR experiments (Gicquel et al., 2013), and the standard deviations were lower than 0.15 threshold cycle (CT). The relative quantifications were obtained as previously described, using the comparative CT (–2^ΔΔCT^) method (Livak and Schmittgen, 2001).

### Statistical Analyses

All experiments are the mean ± SEM of a minimum of three independent experiments. Significances of differences between mean values were assessed using the Student’s *t*-test with significance set as ^∗^*P* < 0.05, ^∗∗^*P* < 0.01.

### Bioinformatics

MEME search was achieved according to Brown et al. (2013).

## Results

### Genomic Context and Transcriptional Analysis of *P. fluorescens tspo* and Pfl01_2810 Encoding a Putative Histidine Kinase

A previous study based on the comparative genome organization of *tspo* and of its flanking genes, revealed that the *tspo* environment was not conserved among bacteria (Chapalain et al., 2009). A *tspo* homolog gene has been predicted in only 7 out of the 48 fully sequenced genomes of *Pseudomonas* (Winsor et al., 2011), among which three are *P. syringae* (pv. *phaseolicola* 1448A, pv. *syringae* B728a, and pv. *tomato* DC3000), and three *P. fluorescens* (strains SBW25, Pf0-1, A506), and in *P. poae* (strain RE^∗^1-1-14). The genomic environment of *tspo* is not conserved among these seven strains, although some degree of conservation exists in each species. For example, in two of the three studied *P. fluorescens* strains, SBW25 and A506, *tspo* forms an operonic structure with an esterase-encoding gene that is involved in lipid metabolism (Winsor et al., 2011). This was, however, not the case for *P. fluorescens* strain Pf0-1. In the latter, a putative histidine kinase encoding gene (Pfl01_2810) was predicted 54 bp upstream of *tspo. In silico* predictions, based on the non-detection of a transcriptional termination signal, suggest that these two genes are included in an operonic structure (**Figure 1A**; Leneveu-Jenvrin et al., 2014). To get further insights into the genetic relations that may exist between the two genes, we investigated their co-transcription by RT-PCR experiments using primers HKF/R, TSPOF/R, HKTSPOF/R designed on Pfl01_2810, or on *tspo*, or on both the two genes, respectively (**Table 2**; **Figure 1A**). A DNA fragment, reflecting the production of mRNA, was observed in each case (**Figure 1B**, lanes 1, 2, and 3), suggesting that the two genes can be co-transcribed in our conditions. A transcriptional fusion, in which a 253 bp fragment laying upstream the +1 translation start site of *tspo* was fused to the *luxCDABE* reporter system (**Figure 1A**, pTSPO), did not reveal any activity when bacteria were grown in LB medium at 28°C (**Figure 1C**, full gray triangles), indicating the absence of promoter in this region. Taken together, our data suggest that Pfl01_2810 and *tspo* are co-transcribed in these conditions, suggesting not only genetic, but also functional links between these two genes in *P. fluorescens* Pf0-1.

### Pattern of *tspO* Transcription during the Growth Course of *P. fluorescens* Pf0-1

To further monitor the transcription of the Pfl01_2810-*tspo* operonic structure, we constructed the pHK-TSPO reporter fusion that contains the 227 bp fragment laying upstream the +1 translational start site of Pfl01_2810 fused to the *luxCDABE* cassette (**Figure 1A**). The activity of pHK-TSPO was followed in LB medium at *P. fluorescens* Pf0-1 optimal growth temperature (28°C) for 24 h. The RL activity resulting from pHK-TSPO was increased by about 1.7-fold in the early exponential phase, and then it decreased rapidly further in the exponential phase (**Figure 1C**, black squares). After 6 h of growth, corresponding to the transition between the late exponential and the entry into the stationary growth phases, the promoter region activity increased slowly but continuously reaching a maximal value of almost 6 × 10^6^ RLU/0.5 s/OD_600_ after 24 h of growth. These data suggest that Pfl01_2810*-tspo* is expressed in a two-step process, i.e., transiently during the early exponential phase, and then, after the transition from exponential to stationary phases, slowly increasing throughout the stationary growth phase. Since the *lux* reporter system requires large amounts of energy and oxygen, we questioned if these growth circumstances might be responsible for the large decrease in promoter activity during the course of growth. We therefore further tested the promoter activity in cells grown in Erlenmeyer flasks (10 ml of culture in 100-ml flasks) under vigorous shaking conditions (180 rpm). When considering the exponential growth phase, the growth and expression patterns were similar in microtiter plates and in Erlenmeyer flasks (**Figures 1C,D**, respectively), showing that the promoter activity during this phase was not due to the specific growth condition imposed by using microtiter plates, but rather reflected decreased transcription of *tspo* during *P. fluorescens* growth, regardless of the aeration status. By contrast, the increase of the promoter activity during the stationary growth phase could not be reproduced in Erlenmeyer flasks, suggesting that this effect would be linked to the cultural conditions. We thus choose to focus our study on the expression pattern of the operonic structure during the exponential growth phase. Taken together, our data indicate that the peak of activity of pHK-TSPO was growth phase dependent and transient, suggesting that the expression of Pfl01_2810 – *tspo* was highly regulated.

### High Salinity and Hyperosmolarity Increase *tspo* Expression

**FIGURE 2:**
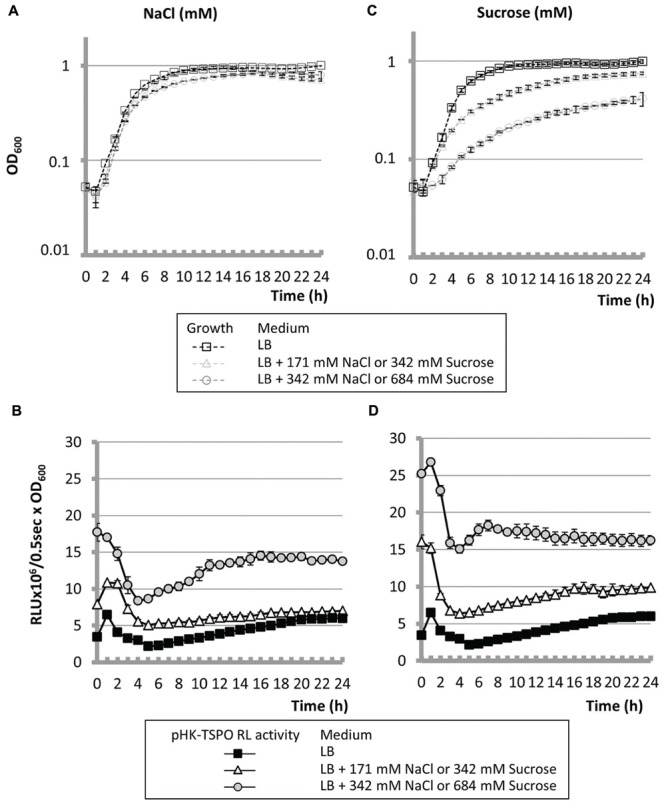
**Hyperosmolarity led to increase Pfl01_2810-tspo transcription.** Growth curves **(A,C)** and transcriptional activity **(B,D)** of *P. fluorescens* Pf0-1 in microtiter wells in LB medium supplemented or not with 171 mM or 342 mM NaCl **(A,B)** or with 342 or 684 mM sucrose **(C,D)**.

In the plant *A. thaliana*, expression of AtTSPO is induced in vegetative tissues by high concentrations of NaCl suggesting that AtTSPO is specifically induced by water-related stress (Guillaumot et al., 2009a,b; Balsemão-Pires et al., 2011). Pfl01_2810 – *tspo* transcription was thus assayed in *P. fluorescens* Pf0-1 grown in LB supplemented with 171 (LB171N) or 342 mM NaCl (LB342N). As shown on **Figure 2A**, the bacterial growth was not deeply affected by the extra supplementation of the LB medium with NaCl. The maximal activities of the promoter region were about 1.7- and 2.9-fold higher during the early exponential growth phase when bacteria were cultured in LB171N or LB342N, respectively, comparatively to LB (**Figure 2B**). The maximal activity reached during the stationary growth phase was similar when bacteria were grown in LB or in LB supplemented with 171 mM of NaCl (LB171N). However, we observed a 2.4-fold increase when we compared the promoter activity in bacteria grown in LB342N and LB at this physiological stage. Taken together, our data suggest that high salinity increased Pfl01_2810 – *tspo* operonic structure expression, as it was the case for AtTSPO (Guillaumot et al., 2009a,b). To assay if salinity or osmolarity was the cause of this increase, we then supplemented LB with sucrose at the same osmolarity as NaCl. LB171N contains 171 mM additional NaCl, i.e., 342 mM osmolytes, since it can dissociate into two ionic species. Addition of 342 and 684 mM sucrose to LB medium led to generate LB342S and LB684S, which have the same osmolarity as LB171N and LB342N, respectively. As shown on **Figure 2C**, increasing the concentration of sucrose led to an increased doubling time, indicating that the presence of sucrose impaired the growth of *P. fluorescens* Pf0-1(doubling times were 1.5- and 2.3-fold increased in LB342S and 684S compared to LB medium, respectively). The bioluminescence patterns in both media were similar to that observed in LB (**Figure 2D**). The maximal activities of the promoter region were 2.6- and 4.3-fold higher during the early exponential growth phase when bacteria were cultured in LB342S or LB684S, respectively, in comparison to LB. The maximal activity reached during the stationary growth phase was increased 1.8- and 2.9-fold in LB342S and LB684S compared to LB (**Figure 2D**). Taken together, our data show that transcription of Pfl01_2810-*tspo* was increased not only in response to high salinity like AtTSPO (Guillaumot et al., 2009a,b), but also in response to hyperosmolarity.

### AlgU is Involved in *tspo* Expression

**FIGURE 3:**
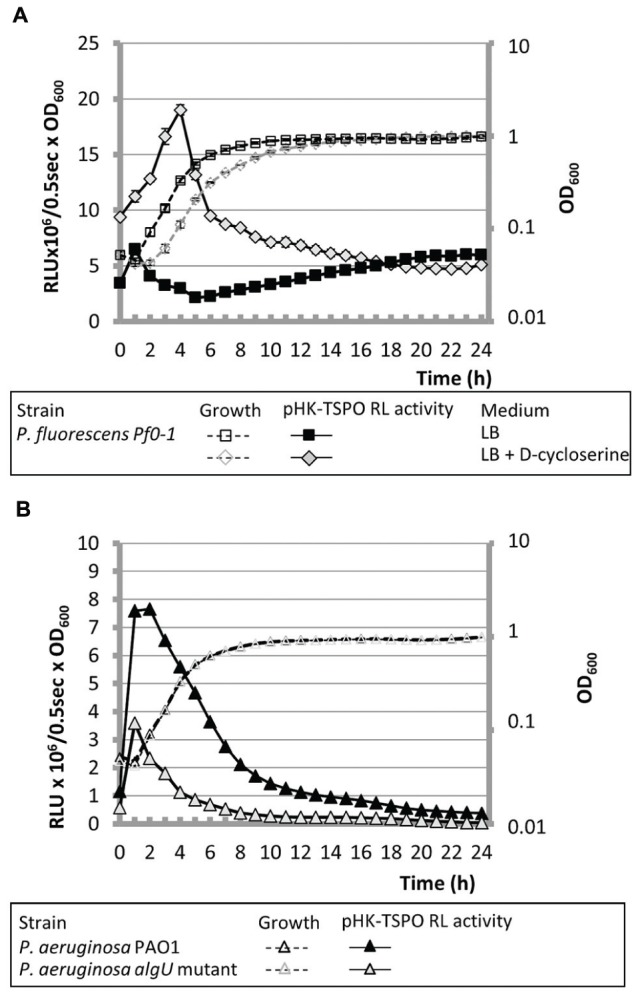
**AlgU is related to the operonic structure transcription. (A)** The growth of *P. fluorescens* Pf0-1 containing pHK-TSPO and the relative bioluminescence levels generated by pHK-TSPO are shown when bacteria were grown in microtiter wells in LB with or without D-cycloserine. **(B)** Growth and relative bioluminescence levels of *P. aeruginosa* PAO1 and its isogenic *algU* mutant containing pHK-TSPO in LB in microtiter wells.

In members of the *Pseudomonas* genus, the response to hyperosmolarity has been shown to be triggered by the master extracytoplasmic function (ECF) sigma factor AlgU (Schnider-Keel et al., 2001). In *P. aeruginosa* mucoid strains, AlgU has been extensively studied due to its involvement in the mucoid conversion, leading to increasing the severity of cystic fibrosis symptoms (Schurr et al., 1996). In non-mucoid strains, AlgU is the major cell wall stress response regulator (Wood et al., 2006). AlgU has been shown to be activated in response to sub-lethal concentrations of D-cycloserine antibiotic leading to peptidoglycan alterations in *P. aeruginosa* (Wood et al., 2006). In an attempt to give further insights into Pfl01_2810 – *tspo* expression, 50 μg.ml^-1^ of D-cycloserine was added to *P. fluorescens* Pf0-1 growth medium. At this sub-lethal concentration, the presence of the antibiotic slightly reduced the growth parameters of *P. fluorescens*, without affecting the final biomass (**Figure 3A**). The presence of D-cycloserine strongly increased the activity of pHK-TSPO during the exponential growth phase. However, the promoter region activity decreased rapidly, without showing an increase during the stationary growth phase, as it was observed after a hyperosmolar treatment. Taken together, these data suggest that the first peak could be linked to the activity of AlgU in our conditions, and that the increase observed during the stationary growth phase under hyperosmolar treatment could be AlgU-unrelated. To get further insight into the role of AlgU in the Pfl01_2810 – *tspo* transcription, the pHK-TSPO reporter fusion and the pAB133 empty vector were transferred into *P. aeruginosa* PAO1 and in its *algU* isogenic mutant strain (Bazire et al., 2010). As in the case of *P. fluorescens* Pf0-1, the promoter fusion activity increased during the early exponential growth phase of wild-type *P. aeruginosa*, but was clearly reduced by more than 2 fold in the *algU* mutant strain (**Figure 3B**). However, no further increase of the fusion activity was observed during the stationary phase in *P. aeruginosa* wild type and *algU* mutant strains, suggesting that this effect might be specific to *P. fluorescens* Pf0-1 and/or to the microtiter growth conditions, as discussed above. Taken together, these data suggest an involvement of AlgU in the operonic structure transcription.

### Expression of Pfl01_2810-*tspo* Operon is Regulated Partly through the Alternative Sigma Factor RpoH

**FIGURE 4:**
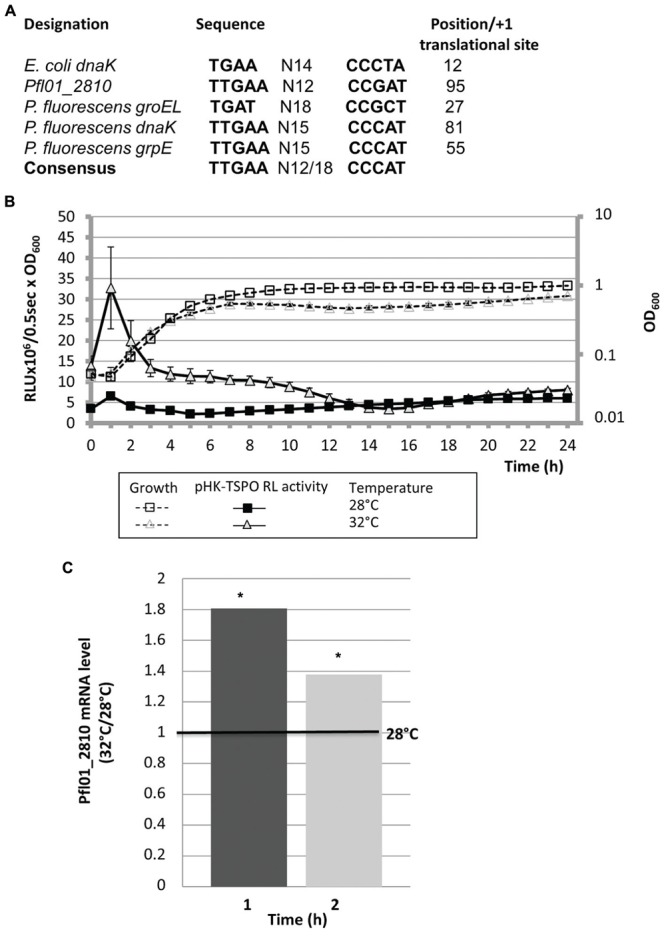
**A putative RpoH-consensus binding site is located in Pfl01_2810*-tspo* promoter region: effect of temperature on growth and TSPO expression. (A)** Sequence alignment of the putative RpoH binding sites identified by MEME in the regions located upstream of *dnaK* in *E. coli* and *groEL, dnaK, grpE*, and Pfl01_2810 in *P. fluorescens* Pf0-1, relatively to the *E. coli* RpoH consensus sequence. Localization of this sequence is given relatively to the +1 translational start site. **(B)** Growth and transcriptional activity of *P. fluorescens* Pf0-1 containing pHK-TSPO reporter fusion in microtiter plates cultured at 28 or 32°C. **(C)** Relative expression of Pfl01_2810 when bacteria were grown at 32°C compared to 28°C by qRT-PCR assays. Significances of differences between mean values were assessed using the Student’s *t*-test with significance set as ^∗^*P* < 0.05.

**FIGURE 5:**
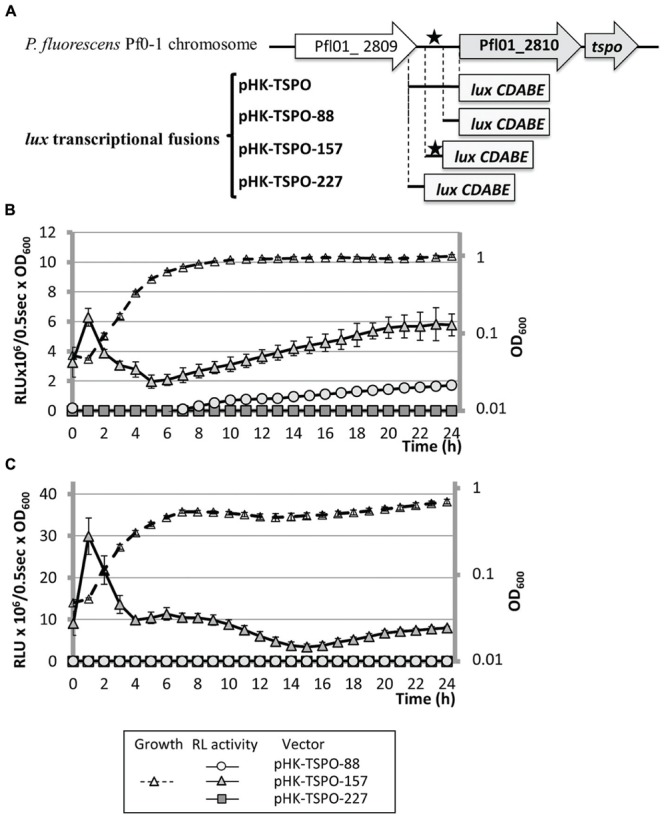
**The activity of the promoter region containing the putative RpoH binding site is increased in response to enhanced growth temperature. (A)** Schematic representation of the four transcriptional fusions. The position of the studied promoter region is indicated relatively to the translational initiation start of each ORF (+1). Putative RpoH binding sequence (black star). Gray bars represent the beginning of the *luxCDABE* reporter cassette of the promoterless pAB133 vector. **(B,C)** Growth in microtiter plates at 28°C **(B)** or 32°C **(C)**, and transcriptional activity of *P. fluorescens* Pf0-1 containing pHK-TSPO-88, pHK-TSPO-157, and pHK-TSPO-227 plasmids.

**FIGURE 6:**
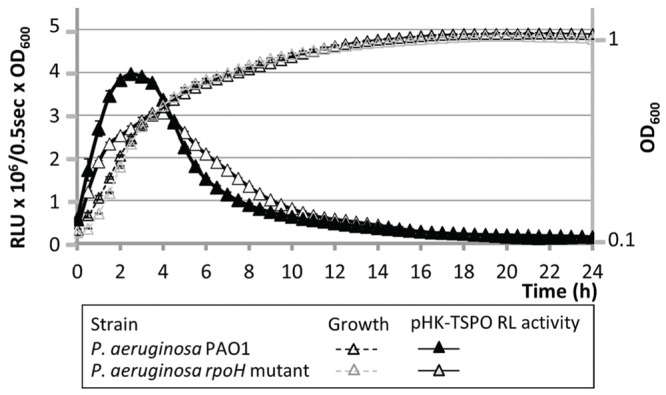
**The activity of the promoter region is lowered in *P. aeruginosa rpoH* mutant strain.** Growth in microtiter plates at 37°C and transcriptional activity of *P. aeruginosa* PAO1 and *rpoH* mutant containing pHK-TSPO-157.

A bioinformatic analysis of the 227 bp region lying upstream of Pfl01_2810-*tspo* using the MEME software (Brown et al., 2013), failed to identify an *algU* consensus binding site (data not shown), suggesting that the effect of AlgU on the expression of this promoter region may be indirect. However, a sequence that showed 90% of identity with the previously proposed binding consensus sequence for the *E. coli* RpoH sigma factor (σ32) was found 95 bp upstream the putative +1 translational start site of Pfl01_2810 (Cowing et al., 1985; **Figure 4A**). This consensus sequence was also detected upstream *P. fluorescens* Pf0-1 *dnaK, groEL*, or *grpE* genes (**Figure 4A**) encoding established direct RpoH targets in *P. aeruginosa* (Potvin et al., 2008) and/or in *E. coli* (Arsene et al., 2000). In these latter bacterial species, RpoH is responsible for the heat-shock response during the upshift of temperature from 30 to 42°C in the early exponential growth phase (Arsene et al., 2000). *P. fluorescens* Pf0-1 is a psychrotrophic strain that is able to grow at temperatures up to 32°C (Silby et al., 2009; Loper et al., 2012). We therefore tested the effect of growth at 32°C on the expression of the operonic structure Pfl01_2810-*tspo*. The promoter activity of the operonic structure was about fivefold increased during the exponential growth phase when bacteria were grown at 32°C compared to 28°C (**Figure 4B**). By contrast, no temperature-related modification could be observed during the stationary growth phase (**Figure 4B**). Pfl01_2810 mRNA expression level was further measured by qRT-PCR experiments on RNAs extracted from bacteria grown at 28 or 32°C. As shown on **Figure 4C**, an increase of the growth temperature resulted in significantly higher levels of expression (about 1.8- and 1.4-fold Pfl01_2810 expression after 1 and 2 h of growth, respectively). Taken together, our data show that the activity of the promoter region laying upstream Pfl01_2810 – *tspo* is increased in response to an elevation of the growth temperature. The identification of an RpoH consensus binding site upstream of Pfl01_2810 is therefore in line with the increased activity of this promoter region at elevated growth temperature. To get further insights into the involvement of RpoH in the temperature-dependent increase of the promoter activity, the transcriptional fusions pHK-TSPO-227, pHK-TSPO-157, pHK-TSPO-88 were constructed, in which three fragments of the studied promoter region were fused to the *luxCDABE* reporter system (**Figure 5A**). Among these three fusions, only pHK-TSPO-157 contained the RpoH putative binding site (**Figure 5A**, black star). The promoter activity of each construct was then assayed when bacteria were grown at 28° (**Figure 5B**) and 32°C (**Figure 5C**). The fusion containing the RpoH-dependent promoter was active at both growth temperatures (**Figures 5B,C**, full triangles), at levels similar to the ones obtained with the large promoter region fusion construct pHK-TSPO (compare with **Figure 4B**), suggesting that the main promoter activity of Pfl01_2810-*tspo* lies in this region. By contrast, no activity was detected at each temperature for the two other constructs during the exponential growth phase. To get further insight into the role of RpoH in Pfl01_2810 – *tspo* transcription, the pHK-TSPO-157 reporter fusion and the pAB133 empty vector were transferred into *P. aeruginosa* PAO1 and in its *rpoH* isogenic mutant strain (Jacobs et al., 2003). *P. aeruginosa* wild type and *rpoH* mutant growth at 37°C was similar. As in the case of *P. fluorescens* Pf0-1, the promoter region activity increased during the growth phase of wild-type *P. aeruginosa* and then decreased. As shown on **Figure 6**, the activity of this promoter region was reduced by about 25% in *P. aeruginosa rpoH* mutant strain, suggesting that RpoH may be at least partly involved in this phenotype.

## Discussion

Based on the Pseudomonas database (Winsor et al., 2011), *P. fluorescens* Pfl01_2810 – *tspo* was predicted to form an operonic structure with the Pfl01_2810 gene encoding a putative hybrid histidine kinase. We confirm in this study that the two genes are indeed co-transcribed, suggesting that both genes are genetically, but also possibly functionally linked. This latter hypothesis is supported by a previous STRING functional analysis (Leneveu-Jenvrin et al., 2014). Histidine kinases are often associated with their response regulator, which contains a receiver or response regulator domain, forming a two component signal transduction system. Hybrid-type histidine kinases comprise a histidine kinase with a receiver domain within one molecule (Stock et al., 2000). It is conceivable that TSPO could participate in the signaling-transduction pathway triggered by Pfl01_2810. Such a functional link between TSPO and a histidine kinase has been previously suggested in other bacteria. For example, in the symbiotic rhizosphere bacterium *Sinorhizobium meliloti*, TSPO has been shown to interact with the sensor FixL of an oxygen-sensing two-component system, regulating nutrient deprivation-induced genes under low oxygen tension (Davey and de Bruijn, 2000). In the photosynthetic bacterium *Rhodobacter sphaeroides*, TSPO has been shown to regulate the expression of bacteriochlorophyll biosynthetic genes by controlling levels of tetrapyrroles in response to the simultaneous presence of light and oxygen (Yeliseev et al., 1997; Yeliseev and Kaplan, 1999, 1995). Little data are actually available about the function of Pfl01_2810 putative histidine kinase. In fact, no Pfl01_2810 homolog gene was predicted in the sequenced genomes of *Pseudomonas fluorescens* strains (http://www.pseudomonas.com, Winsor et al., 2011), suggesting that the association between Pfl01_2810 and *tspo* was specific to *P. fluorescens* Pf0-1. A Pfl01_2810 homolog was, however, predicted in 22 sequenced genomes of *Pseudomonas* species, among which *P. putida* (nine strains), *P. syringae* (six strains), *P. stutzeri* (five strains), *P. entomophila* (one strain), and *P. poae* (one strain). In these strains, the Pfl01_2810 homolog gene is often clustered with a (GntR-, LysR- or TetR-like) transcriptional regulator, and with a cytochrome *c* oxydase or an iron–sulfur cluster binding protein encoding gene in case of *P. putida* strains, or a cation transporter encoding gene in case of *P. stutzeri* and some *P. syringae* strains (Winsor et al., 2011). Taken together, these data may suggest a link between Pfl01_2810 and the oxidative level homeostasis, that should be investigated in future studies.

A transcriptional fusion approach was used herein to get further insights into *tspo* expression in *P. fluorescens* Pf0-1. We show that Pfl01_2810 – *tspo* cluster transcription was growth-phase dependent and transient, since a peak was observed in the early exponential phase when bacteria were grown in LB medium at 28°C. These data raised the possibility that TSPO may only be temporarily required by the cells during the exponential growth phase and hence possibly rapidly down-regulated after induction, suggesting that expression of these two genes was tightly regulated. Accordingly, TSPO was shown to be transiently induced in the Arabidopsis plant cell where it is one of the most strongly induced “early” genes (Kreps et al., 2002; Seki et al., 2002; Zimmermann et al., 2004; Winter et al., 2007; Guillaumot et al., 2009a; Hermans et al., 2010; Vanhee and Batoko, 2011; Vanhee et al., 2011). Similar transient expression of *tspo* has been reported in *Physcomitrella patens* following chitosan treatment leading to the generation of an oxidative stress. In this moss plant, *tspo* expression was indeed induced by four- to fivefold about 15 to 30 min following treatment, and then decreased rapidly about 90 min after treatment (Lehtonen et al., 2012).

In plants, AtTSPO has been suggested to participate to the global plant water stress response, since its level increases in plants producing ABA, the phyto-hormone that regulates plant water status through regulation of stomatal closure (Finkelstein et al., 2002; Nambara and Marion-Poll, 2005), *tspo* being one of the ABA-responsive genes (Guillaumot et al., 2009a,b; Balsemão-Pires et al., 2011). Here we demonstrate that growth of *P. fluorescens* Pf0-1 in high salinity and osmolarity conditions leads to an increase in the operonic structure transcription, at least partly through activity of AlgU. AlgU is the major ECF sigma factor that is involved in maintaining the cell wall integrity and triggers the global response to cell wall damages in *Pseudomonas* species (Wood and Ohman, 2009). For example, it has been shown that in *P. protegens* CHAO, AlgU favors bacterial resistance to dessication and osmotic stress (Schnider-Keel et al., 2001). Taken together, our results suggest that Pfl01_2810-*tspo* operon is part of the cell wall stress response triggered by AlgU. Through a bioinformatic study using MEME algorithm, we could, however, not detect an AlgU binding motif, suggesting that the effect of AlgU on Pfl01_2810 – *tspo* expression could be indirect. However, a sequence that presents a high level of similarity with the binding site for the alternative sigma factor RpoH was detected in this region, suggesting that RpoH could be involved directly in the operonic structure transcription. This hypothesis is supported by (i) the increased transcription of the operonic structure when bacteria are grown at high temperature, (ii) the increased activity of the reporter fusion containing the RpoH putative binding motif, but not of the other ones in which this region was absent, (iii) the reduced promoter activity in a *P. aeruginosa rpoH* mutant strain, and finally, (iv), the fact that RpoH itself is part of AlgU regulon, since at least one of its identified promoters was AlgU-dependent (Schurr et al., 1996; Keith and Bender, 1999). However, the promoter activity was reduced by only 25% in *P. aeruginosa rpoH* mutant, suggesting that RpoH was not the main actor in the promoter activity, at least in *P. aeruginosa*. *In vitro* electrophoretic mobility gel-shift assay or yeast-one hybrid assay should now be used to confirm the binding of RpoH to its predicted promoter site. Taken together, these data suggest that Pfl01_2810-*tspo* is mainly regulated by AlgU, and to a lower extent by RpoH, through a direct or indirect molecular mechanism. Interestingly, two alternative sigma factors RpoH(I) and RpoH(II) were identified in *R. sphaeroides*, RpoH(I) being mainly involved in the reactive singlet oxygen response, and RpoH(II) in the heat stress response. Through a global predictive study, Nuss et al. (2010) identified a conserved binding sequence for RpoH(II) upstream *R. sphaeroides tspo.* Interestingly, in this bacterium, RpoH(II) is controlled by RpoE, the AlgU homolog ECF sigma factor in *R. sphaeroides* (Nuss et al., 2010; Dufour et al., 2012). The link between RpoH and *tspo* is further strengthened by the global transcriptomic study performed on the symbiotic bacterium *Synorhizobium meliloti* during heat shock and stationary phase growth (Barnett et al., 2012). These bacteria possess two RpoH-like sigma factors, i.e., RpoHI that is the major player in the heat shock response, and RpoHII that is mainly involved during the stationary growth phase. Interestingly, the *tspo* gene has been reported to be induced in a RpoH1-dependent manner (Supplementary Table S1 in Barnett et al., 2012). Taken together, our data support that the operonic structure Pfl01_2810 – *tspo* belongs to the AlgU/RpoH regulons.

RpoH and AlgU are multifaceted stress response regulators that maintain protein and membrane functionality homeostasis, and also sense imbalances in membrane proteins (Nonaka et al., 2006; Rhodius et al., 2006). In addition to impacting protein structures, higher temperatures and hyperosmolarity result in increased membrane fluidity. In Mammals, TSPO binds cholesterol, leading at least partly, to modulation of the mitochondrial membrane fluidity and permeability (Papadopoulos et al., 2006), and in plants TSPO is a water stress-responsive protein (Guillaumot et al., 2009a,b) that has been recently involved in the regulation of cell surface expression of an aquaporin (Hachez et al., 2014). Taken together, our data strongly supports the hypothesis that TSPO might be part of the cellular stress response network in *P. fluorescens* Pf01.

## Author Contributions

Conceived and designed experiments: EB, NC, CL-J, SC. Performed the experiments: CL-J, EB, OM. Analyzed the data: CL-J, EB, NC, SC. Wrote the paper: CL-J, PC, NC, MF, SC. All authors read and approved the final manuscript.

## Conflict of Interest Statement

The authors declare that the research was conducted in the absence of any commercial or financial relationships that could be construed as a potential conflict of interest.
